# Revisiting oxidation and reduction reactions for synthesizing a three-dimensional hydrogel of reduced graphene oxide†

**DOI:** 10.1039/d4ra05385k

**Published:** 2024-09-27

**Authors:** Hon Nhien Le, Thi Bang Tam Dao, Trung Do Nguyen, Duc Anh Dinh, Chi Nhan Ha Thuc, Van Hieu Le

**Affiliations:** a Faculty of Materials Science and Technology, University of Science 227 Nguyen Van Cu Street, Ward 4, District 5 Ho Chi Minh City 700000 Vietnam lhnhien@hcmus.edu.vn htcnhan@hcmus.edu.vn lvhieu@hcmus.edu.vn; b Multifunctional Materials Laboratory, University of Science Ho Chi Minh City 700000 Vietnam; c Vietnam National University Linh Trung Ward, Thu Duc City Ho Chi Minh City 700000 Vietnam; d NTT Hi-Tech Institute, Nguyen Tat Thanh University Ho Chi Minh City 700000 Vietnam

## Abstract

An improvement to Hummers' method involving a cascade-design graphite oxidation reaction is reported to optimize safety and efficiency in the production of graphite oxide (GrO) and graphene oxide (GO). Chemical reduction using highly alkaline ammonia solution is a novel approach to synthesizing reduced graphene oxide (RGO). In this original research, we revisit the oxidation and reduction reactions, providing significant findings regarding the synthetic pathway to obtain a bioinspired water-intercalated hydrogel of RGO nanosheets. Influential factors in the graphite oxidation reaction, typically the exothermic reaction temperature and hydrogen peroxide effect, are described. Furthermore, the chemical reaction of GO reduction using highly alkaline ammonia solution (pH 14) was investigated to produce hydrated RGO nanosheets assembled in a hydrogel structure (97% water). Three-dimensional assembly and water intercalation are key to preserve the non-stacking state of RGO nanosheets. Therefore, ultrasound transmission to aqueous channels in the macroscopic RGO hydrogel vibrated and dispersed the RGO nanosheets in water. Analytical results revealed the single-layer nanostructures, functional groups, optical band gaps, optimized C/O ratios, particle sizes and zeta potentials of GO and RGO nanosheets. The reversible self-assembly of RGO hydrogels is essential for many applications, such as RGO coatings and polymer/RGO nanocomposites. In a water purification application, the RGO hydrogel was dispersed in aqueous solution by simple agitation and showed a high capacity for organic dye adsorption. After the adsorption, the RGO/dye particles were easily removed by filtration through ordinary cellulose paper. The process of adsorption and filtration is effective and inexpensive for practical environmental remediation. In summary, a bioinspired structure of RGO hydrogel is conceptualized for prospective nanotechnology.

## Abstract 2

A cascade-design self-heating oxidation reaction provides outstanding energy and chemical efficiencies for graphene oxide production. A reduction reaction using highly alkaline ammonia leads to a bioinspired hydrogel of reduced graphene oxide.

## Graphical abstract

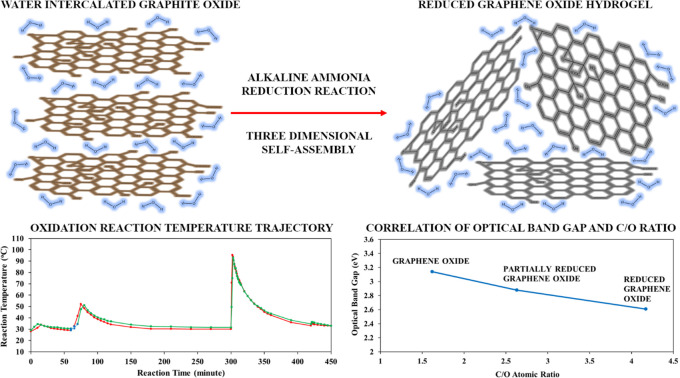

## Introduction

Oxidation and reduction reactions are two sequential prime steps in the synthetic pathway of converting graphite into graphene oxide (GO) and reduced graphene oxide (RGO). In 1859, Benjamin Collins Brodie prepared a mixture of potassium chlorate (KClO_3_) and fuming nitric acid (HNO_3_) for the oxidation of graphite at 60 °C for a few days, which resulted in graphite oxide (GrO) for the first time.^1,2^ In 1898, L. Staudenmaier improved the oxidation procedure using a smaller proportion of fuming nitric acid and the additional reagent sulfuric acid (H_2_SO_4_). The main oxidant potassium chlorate was slowly added in small doses, making the reaction safer.^3^ In 1937, the graphite oxidation reaction mixture was modified by V. L. Hofmann, who used potassium chlorate, concentrated nitric acid and concentrated sulfuric acid as oxidizing reagents.^4^ With its good degree of graphite oxidation, the Hofmann method was considered to be an alternative approach to synthesizing GrO.^5,6^ In 1909, the combination of potassium permanganate (KMnO_4_) and sulfuric acid for the graphite oxidation reaction was first described by G. Charpy.^7,8^ In 1958, William S. Hummers and Richard E. Offeman reported the scalable preparation of graphitic oxide using the reagents potassium permanganate, sulfuric acid, sodium nitrate (NaNO_3_) and hydrogen peroxide (H_2_O_2_).^9^ The classical Hummers' method that initially produced GrO with a C/O ratio of 2.25 is the most well-known synthesis of GrO today. The paper by William S. Hummers *et al.* has been cited more than 26 000 times in the scientific literature.

About 50 years later, the discovery of graphene's excellent properties and the chemical synthesis of graphene-based nanosheets from GrO initiated great interest in investigating graphite oxidation reactions.^7,10,11^ In the early period of the 2000s, the classical Hummers' method was mostly conducted for synthesizing multilayer GrO, which was subsequently sonicated in water to produce GO nanosheets.^12–14^ It is notable that the Hummers' method used a graphite : KMnO_4_ ratio of 1 : 3 (w/w) and a graphite : NaNO_3_ ratio of 1 : 0.5 (w/w). In 2010, D. C. Marcano and colleagues modified the reaction mixture using a graphite : KMnO_4_ ratio of 1 : 6 (w/w) and an H_2_SO_4_/H_3_PO_4_ mixture of 9 : 1 (v/v).^15^ In 2013, a paper by J. Chen *et al.* reported an improved Hummers' method that kept the graphite : KMnO_4_ ratio of 1 : 3 (w/w) and excluded NaNO_3_ (because of the risk of evolving toxic NO_2_ gas in the reaction).^16^ Additionally, it is crucial that the mechanisms of graphite oxidation reactions were investigated and elucidated, as in the papers by T. Nakajima *et al.*,^17,18^ S. Pan *et al.*,^19^ A. M. Dimiev *et al.*^20,21^ and N. Morimoto *et al.*^22^ A. M. Dimiev and colleagues also demonstrated that water plays an important role in the graphite oxidation reaction, being responsible for C–O bonding of hydroxyl groups on the graphite basal planes.^23^

The research and development of GrO synthesis has increased significantly for industrialization and commercialization in recent years. In large-scale production, reaction safety and process efficiency are principal challenges.^24–27^ In Hummers' approaches, the dissolution of KMnO_4_ in H_2_SO_4_ generates the powerful oxidant Mn_2_O_7_, also known as MnO_3_^+^ or Mn(vii) compound, which is an explosive at temperatures above 55 °C. During the graphite oxidation process, there are several exothermic reactions that may cause thermal runaway and explosive risks. P. Lakhe *et al.* reported that the dissolution and reaction of KMnO_4_ in the mixture of graphite and H_2_SO_4_ is a thermal runaway process that could increase the reaction temperature above the safety limit of 55 °C.^28^ The step of adding water to the reaction mixture is a much more exothermic and time-consuming process. Thermal runaway in which the reaction temperature increases to the explosive limit has been a principal safety issue in the chemical industry.^29^ To improve safety and efficiency in GrO production technology, our recent study described a cascade-design graphite oxidation reaction that harnesses exothermic energy for the self-heating reaction and optimizes the reaction time and chemical ratios, with a graphite : KMnO_4_ ratio of 1 : 2 (w/w), graphite : H_2_SO_4_ ratio of 1 : 30 (w/v) and water : H_2_SO_4_ ratio of 2.4 : 1 (v/v).^30^

In the area of the chemical reduction of GO nanosheets, since the early research period of the 2000s, hydrazine hydrate (N_2_H_4_) has been used as a potent reagent to obtain RGO nanosheets with a high reduction degree.^31,32^ Other effective reducing agents include sodium borohydride (NaBH_4_), ascorbic acid (C_6_H_8_O_6_), hydroiodic acid (HI), metallic catalysts (Al, Fe, Zn and TiO_2_) and other organic reagents.^33–37^ Reduction reactions remove the oxygen-containing functional groups and restore the conductive π-conjugated networks in graphene-based nanosheets; however, RGO nanosheets tend to agglomerate in an aqueous environment due to their hydrophobic nature and the π–π interaction force. Since the intersheet distance is closer and the surface area of close–distance interaction is sufficiently larger, van der Waals potential of graphene stacking is as high as the irreversible level.^38^ The restacking of graphene-based nanosheets causes the drawbacks of low aqueous dispersibility and accessible surface area. Intensive research has elucidated that electrostatic repulsion, surface hydration, nanoparticle intercalation and three-dimensional assembly are useful strategies for preventing the irreversible van der Waals stacking of graphene nanosheets.^39–47^ Typically, our research articles have reported the reversible self-assembly of GO–ZnO and RGO/SnO_2_ hydrogels.^48,49^ Hydrated metal oxide nanoparticles and water molecules on graphene nanosheets increase the interlayer distance and decrease the stacked surface area, facilitating the ultrasonic exfoliation of the macroscopic hydrogels into graphene-based nanostructures.

In this manuscript, the cascade design of the graphite oxidation reaction is revisited to provide significant findings on the reaction temperature trajectory and hydrogen peroxide effect. Moreover, GO nanosheets synthesized using the cascade procedure were chemically reduced using the green method of highly alkaline ammonia solution. After the reduction reaction, the RGO nanosheets were self-assembled into a supramolecular hydrogel structure. The oxygen-containing functional groups remaining on the RGO nanosheets are hydrophilic to retain hydration layers as well as water intercalation among the RGO nanosheets in the three-dimensional hydrogel. Therefore, the macroscopic RGO hydrogel has the extraordinary advantage of good dispersibility in water, especially with the support of ultrasound treatment. The exfoliated nanostructures in water are RGO nanosheets with a narrowed band gap energy and optimized C/O ratio. The as-synthesized RGO hydrogel was utilized for organic dye adsorption in a simple and effective water purification process. The RGO hydrogel provided high adsorption capacity and facile removal by filtration through ordinary cellulose paper. The water purification process has great potential for industrial and household applications.

## Experimental methods

### Materials

Natural graphite (Shanghai Zhanyun Chemical Company) and potassium permanganate 99.5% (Duc Giang Chemicals Group) were used in the synthesis of graphite oxide. Sulfuric acid 96%, hydrogen peroxide 30%, hydrochloride acid 36% and ammonia solution 25% were obtained from Xilong Chemical Company. Methylene blue trihydrate (≥98.5%) was purchased from Xilong Scientific Company. Cellulose filter paper (diameter of 9 cm, UNI-Sci) and distilled water were employed in the experiments.

### Synthesis of graphite oxide and graphene oxide

Natural graphite was converted into graphite oxide (GrO) using the cascade design oxidation reaction with Mn(vii) compound.^30^ Hummers' reagents, including KMnO_4_, 96% H_2_SO_4_ and water, were used with optimized ratios in the synthetic procedure. First, 10 g KMnO_4_ was dissolved in 100 mL of 96% H_2_SO_4_ to obtain a solution of Mn(vii)/H_2_SO_4_. In the first cascade step, the suspension of 5 g graphite in 50 mL of 96% H_2_SO_4_ was poured slowly into the as-prepared Mn(vii)/H_2_SO_4_ solution. During the first cascade step, the glass reactor was jacketed by an ambient water bath (water temperature ∼28 °C) for different cooling times (5 minutes and 10 minutes). After the cooling, the reactor was placed at ambient room conditions (∼34 °C) and magnetically stirred for nearly 4 hours. An infrared thermometer was used to record the reactor temperatures during the exothermic cascades to ensure safe reaction temperatures below the limit of 55 °C. In the second cascade step, the mixture of graphite/Mn(vii)/H_2_SO_4_ was gently poured into 360 mL water under stirring in a glass beaker. The chemical reactions quickly generated high exothermic energy for the self-heating oxidation reaction (peak temperature >90 °C). After 2 hours, the reaction temperature decreased to near room conditions. 5% H_2_O_2_ solution was added at the end of the oxidation reaction process. Different amounts of 5% H_2_O_2_ solution (150 mL in procedure 1, 90 mL in procedure 2 and 30 mL in procedure 3) were used for investigating the hydrogen peroxide effect on the GrO product and GO nanosheets. After approximately 1 day of stirring with H_2_O_2_ solution, the GrO particles were sedimented and washed thoroughly with 5% HCl solution and pure water, followed by drying and grinding.

(1) Graphite + H_2_SO_4_ → H_2_SO_4_-intercalated graphite.

(2) KMnO_4_ + 2H_2_SO_4_ → Mn_2_O_7_ + H_2_O + 2 KHSO_4_ (Δ*H* < 0) Mn_2_O_7_ + 2H_2_SO_4_ → 2[MnO_3_]^+^[HSO_4_]^−^ + H_2_O (Δ*H* < 0) (Mn_2_O_7_ or MnO_3_^+^ is Mn(vii) compound, which is explosive at temperatures above 55 °C).

(3) H_2_SO_4_-intercalated graphite + Mn(vii) → Mn(vii)/H_2_SO_4_-intercalated graphite (Δ*H* < 0).

(4) Graphite/Mn(vii)/H_2_SO_4_ + H_2_O → oxidized graphite + H_2_SO_4_ + Mn(iv) + O_3_ (Δ*H* < 0).

(5) 2Mn_2_O_7_ + 2H_2_O_2_ + 4H_2_SO_4_ → 4MnSO_4_ + 6H_2_O + 2O_3_ + 3O_2_ (Δ*H* < 0).

To prepare the GO nanosheets, 2.5 g of the obtained GrO powder was ultrasonically dispersed in 500 mL water for about 45 minutes. Ammonia solution was used to adjust the pH of the mixture 10, stabilizing the GO nanosheets through electrostatic repulsion. After one day of natural sedimentation, the supernatant GO solution was collected. The concentrated GO solution was used for the subsequent analysis and chemical reaction.

### Synthesis of reduced graphene oxide hydrogel

First, 450 mL concentrated GO solution was mixed with 25% ammonia solution to obtain an ammonia concentration of ∼2.5% (pH 14) for the reduction reaction. The glass reactor was covered to prevent ammonia evaporation and heated to about 90 °C without agitation. Different reaction times at 90 °C (2 hours and 3 hours) were applied to produce partially reduced graphene oxide (pGO) and reduced graphene oxide (RGO) respectively. During the reduction reaction, the GO nanosheets were gradually reduced and self-assembled into three-dimensional agglomerates. After cooling to room temperature, the suspension was filtered through a cellulose paper to collect a black slurry, followed by washing with pure water until the filtrate pH was ∼8. The RGO slurry was dried at 60 °C for about 4 hours, producing a macroscopic RGO hydrogel (moisture ∼97%).

### Water purification experiment

Methylene blue trihydrate powder (MB) was dissolved in water and diluted to obtain a concentrated MB solution of 40 ppm. Different amounts of RGO hydrogel (97% water and 3% RGO) were used for the treatment of 200 mL of the 40 ppm MB solution. Typically, 0.4 g RGO hydrogel (containing 0.012 g RGO) was dispersed in 200 mL of 40 ppm MB solution using magnetic agitation. After certain time periods (15, 30, 45, 60, 90, 120 and 1440 minutes), 15 mL of the suspension was collected and centrifuged at 4000 rpm for 5 minutes to remove the solid. The collected solutions were analyzed using a spectrophotometer (Agilent Cary 60) to determine the MB concentrations. The intensities of the absorption peak at 665 nm were used to establish the calibration curve and calculate the MB concentration. The experiments were repeated three times. The MB adsorption capacity was calculated based on the amount of adsorbed MB and the weight of RGO in the adsorption process.

After the stage of organic dye adsorption, the MB/RGO suspension was filtered through an ordinary cellulose paper (pore size of 15–20 μm) to remove solid particles. The water obtained after the filtration was analyzed using ultraviolet-visible absorption spectroscopy to evaluate the amount of MB remaining as well as the water purity. The flow rate of the filtration process (L m^−2^ h^−1^) was also calculated using the filtration time, filtrate weight and filter paper area.

### Analytical methods

An analytical balance (Ohaus Pioneer) and a moisture analyzer (A&D MX-50) were used for measurement of the weight and moisture content of powder and hydrogel materials. The water temperature and reaction temperature were recorded with the support of an infrared thermometer (Benetech GM-320). X-ray diffraction (XRD) patterns of the prepared materials were characterized using a Bruker D2 Phaser X-ray diffractometer instrument (Cu Kα radiation source with an incident light wavelength of 1.54 Å). Fourier transform infrared spectroscopy (FTIR) was conducted using an FT/IR-4700 type-A spectrometer. A UP100H (Hielscher) ultrasonicator was used in ultrasound processes for the exfoliation and dispersion of nanostructures in water medium. Ultraviolet-visible absorption spectroscopy (UV-vis) of RGO and GO-ZnO nanomaterials was conducted using a spectrophotometer (Jasco V-670). The Tauc plot method was applied in the calculation of the optical band gap energy using the equation:(*αhν*)^*n*^ = *A*(*hν* − *E*_g_)where *α* is the optical absorption coefficient, *hν* is the incident photon energy, *n* = 2 for a permissible direct transition or *n* = ½ for a permissible indirect transition, and *A* is a characteristic number. It was demonstrated that the absorption coefficient *α* = 2.303 *A*_λ_/*z*, where *A*_λ_ is the light absorbance and *z* is the optical path length in the UV-vis spectroscopic analysis.

A JEOL JSM-IT200 scanning electron microscopy (SEM) and energy dispersive X-ray spectroscopy (EDS) system was employed for integrative analysis of morphology, nanostructure, elemental composition and mapping. Samples of RGO nanomaterials were coated with a platinum layer before SEM–EDS analysis. Aqueous dispersions of graphene-based nanosheets in water (concentration of 7 ppm) were prepared for electron transmission microscopy (TEM), particle size analysis and zeta potential measurement. The nanostructures of GO and RGO nanosheets were imaged using a TEM instrument (JEM-1010, JEOL). Particle size distribution and zeta potential analysis of GO and RGO nanosheets were conducted using a Zetasizer Ultra (Red Label) instrument (Malvern Panalytical). The particle size distributions were analyzed using the setting with a horizontal filter (for non-spherical particle shape).

## Results and discussion

### Exothermic cascades and hydrogen peroxide effects in the graphite oxidation reaction

**Fig. 1:**
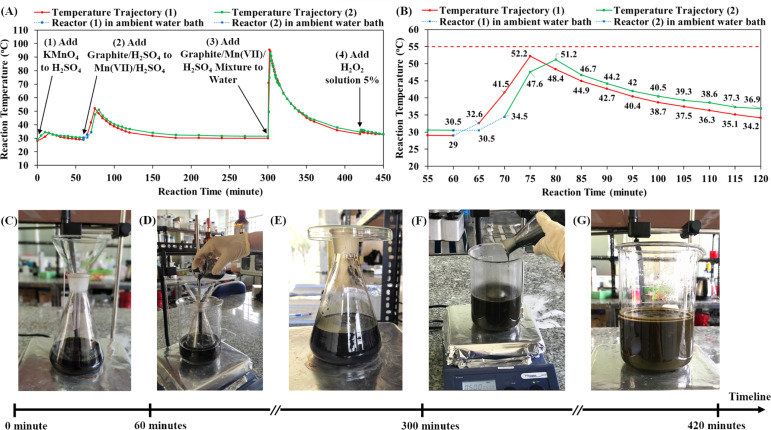
(A) Temperature trajectory in the graphite oxidation process, specifically, the exothermic reactions of dissolving KMnO_4_ in H_2_SO_4_ solution (1), adding graphite/H_2_SO_4_ mixture to Mn(vii)/H_2_SO_4_ solution (2), adding graphite/Mn(vii)/H_2_SO_4_ to water (3) and adding H_2_O_2_ solution 5% to the reaction (4). (B) Expanded view of the temperature trajectory in the first cascade step, including periods during which the reactor was in an ambient water bath and at ambient room conditions. (C) Mn(vii)/H_2_SO_4_ solution after dissolving KMnO_4_ in 96% H_2_SO_4_. (D) Graphite/H_2_SO_4_ mixture was poured into Mn(vii)/H_2_SO_4_ solution (the reactor in an ambient water bath). (E) Mixture of graphite/Mn(vii)/H_2_SO_4_ under stirring (the reactor in the ambient room environment). (F) Graphite/Mn(vii)/H_2_SO_4_ mixture was gently poured into water. (G) Brown suspension of oxidized graphite before adding 5% H_2_O_2_ solution.

Our previous paper presented a cascade design for the graphite oxidation reaction, which includes two main cascade steps: the first cascade of the graphite/H_2_SO_4_ mixture into the Mn(vii)/H_2_SO_4_ mixture and the second cascade of graphite/Mn(vii)/H_2_SO_4_ mixture into water.^30^ Both cascade steps generate significant exothermic energy for self-heating reactions, which is an advantage for large-scale production. However, it is crucial to investigate the reaction temperature profiles in order to control thermal runaway processes in industrial chemical factories.^29,50^ Herein, the temperature trajectories during the graphite oxidation reaction were recorded in detail using an infrared thermometer (Fig. 1A and B). Accordingly, there are four exothermic processes in the synthetic route. First, the dissolution of KMnO_4_ in concentrated H_2_SO_4_ over at least 10 minutes increased the reactor temperature from ∼29 °C to ∼34 °C and prepared the mixture of Mn(vii)/H_2_SO_4_ (Fig. 1C). After 60 minutes (*t* = 60 minutes), the first cascade step was conducted by slowly pouring the suspension of graphite/H_2_SO_4_ into the mixture of Mn(vii) compound (Fig. 1D). The exothermic reaction in the first cascade process may cause thermal runaway that potentially increases the reaction temperature above the safety limit of 55 °C. Thus, the reactor was placed in an ambient water bath (water temperature of 28 °C) for cooling and to control the thermal runaway process.

In the first cascade step, the reaction between the graphite particles and Mn(vii) compound was energetic, causing temperature increase and thermal runaway. Two synthetic experiments were conducted in which the reactor was cooled in the ambient water bath for different lengths of time (5 minutes and 10 minutes). When the reactor was placed in ambient room conditions (Fig. 1E), the reaction temperature began to rise considerably. As shown in Fig. 1B, when the cooling time of 5 minutes was used, the reaction temperature increased to a peak of 52.2 °C, and with the cooling time of 10 minutes, the peak reaction temperature was 51.2 °C. These temperature maxima were below the safety limit of 55 °C, but the cooling time of 10 minutes was preferred for controlling the risk of thermal runaway. The total heat released from the exothermic process was calculated to be 19.19 KJ in reaction (1) and 17.12 KJ in reaction (2) (see ESI Fig. S1†). The longer cooling time in reaction (2) dissipated a greater amount of exothermic energy, resulting in a decrease of by 10.79% in the total exothermic heat compared to reaction (1). Additionally, it is noted that the exothermic reaction did not occur immediately; the reaction temperature increased gradually from room level to the peak. When graphite particles were dispersed in the Mn(vii)/H_2_SO_4_ solution, the Mn(vii) oxidizing agents diffused and reacted with graphite. It was demonstrated that in the reaction the Mn(vii) intercalated in the layered structures of graphite.^21,22^ A. G. Bannov *et al.* showed that the graphite oxidation reaction by Mn(vii) occurred even at low temperature (∼0 °C) and became much stronger at 35 °C.^51^ In this study, the exothermic energy released from the oxidation reaction gradually increased the reaction temperature. Higher temperature accelerated the oxidation reaction as well as the exothermic process. The thermal runaway raised the reaction temperature from 34.5 °C to 51.2 °C after 10 minutes under ambient room conditions. After reaching its peak, the reaction temperature declined slowly to room temperature (∼36.9 °C at *t* = 120 minutes, Fig. 1B). The exothermic energy of the oxidation reaction between graphite and Mn(vii) compound was utilized for self-heating, efficient and safe synthesis. The first cascade step is important to form the intermediate structure of Mn(vii)/H_2_SO_4_-intercalated graphite.

In the second cascade step (starting at *t* = 300 minutes), the mixture of graphite/Mn(vii)/H_2_SO_4_ (Mn(vii)/H_2_SO_4_-intercalated graphite) was poured into a beaker of water under stirring (Fig. 1F). During the strong exothermic cascade, the reaction temperature reached 49.5 °C after 1 minute, 75 °C after 2 minutes and the peak of 93 °C after 3 minutes (*t* = 303 minutes). The cascade step was finished in only 3 minutes. The reaction temperature then decreased to 91 °C (at *t* = 304 minutes) and 87.3 °C (at *t* = 305 minutes). It is the hydration of concentrated sulfuric acid in water that generated the energetic heat (H_2_SO_4_ + H_2_O → H_3_O^+^ + HSO_4_^−^). As described in the previous study, the high temperature is beneficial to the graphite oxidation reaction by Mn(vii) and water molecules.^30^ In concert with the electrophilic centers of the Mn(vii), water molecules diffused into graphite galleries and formed hydroxyl groups bonding to the graphite basal planes. The strong oxidation reaction made the suspension turn brown, which was attributed to the color of graphitic oxide (Fig. 1G). The exothermic reaction between H_2_SO_4_ and water occurred very fast and reached a peak temperature of ∼93 °C in just 3 minutes. Then, it took 1 hour for the suspension temperature to decrease to 43.7 °C (at *t* = 360 minutes) and 2 hours for the suspension to cool to 34.5 °C (at *t* = 420 minutes).

Notably, the addition of 5% H_2_O_2_ solution to the reaction suspension is also an exothermic process, raising the reaction temperature from 34.5 °C (at *t* = 420 minutes) to 36.2 °C (at *t* = 421 minutes). The role of the reagent H_2_O_2_ was to terminate the oxidation reaction of manganyl compounds. Additionally, H_2_O_2_ is also an effective oxidizing agent that may account for additional oxidation reactions on graphite oxide. In fact, the addition of 5% H_2_O_2_ solution created bubbles (probably O_2_ evolution) in the GrO suspension for a few days. According to Table 1, the investigation of the amount of H_2_O_2_ showed that the use of a higher amount of 5% H_2_O_2_ solution led to a higher solid yield of GrO product and wider band gap of GO nanosheets. H_2_O_2_ helped to increase the amount of oxygen-containing functional groups on the GO nanosheets as well as the solid yield of GrO product. As the oxidation degree of the GrO became higher, the π-conjugated sp^2^-carbon networks in the GO nanosheets further disintegrated, resulting in widening of the GO band gap (3.14 eV for procedure 1 in which 150 mL H_2_O_2_ 5% was used). In previous research, the influence of the H_2_O_2_ : H_2_SO_4_ mixture (volumetric ratio of 1 : 15) on the graphite oxidation reaction at room temperature was reported, revealing the oxidation effect on the graphite structure.^52^ Other papers have reported that hydroxyl radicals derived from H_2_O_2_ are responsible for additional oxygen-containing functional groups on GO nanosheets.^53,54^ Therefore, having an appropriate H_2_O_2_ concentration in the reaction mixture provides a positive effect on the graphite oxidation process.

**Table 1:** Relationship between the amount of 5% H_2_O_2_ solution and the solid yield of GrO and optical band gap of GO

	Volume of H_2_O_2_ solution 5% (mL)	Solid yield of GrO powder (%)	Optical band gap of GO nanosheets (eV)
Procedure 1	150	132	3.14
Procedure 2	90	127	3.0
Procedure 3	30	123	2.9

### Synthesis of reduced graphene oxide hydrogel using highly alkaline ammonia solution

**Fig. 2:**
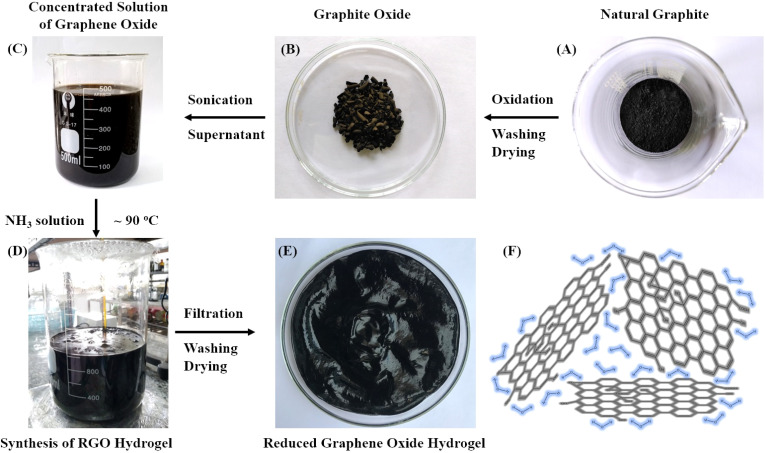
Synthetic scheme from natural graphite (A) to GrO (B), concentrated GO solution (C), chemical reduction reaction (D), and RGO hydrogel (E). (D) Chemical reduction of GO nanosheets using ammonia solution at ∼90 °C. (E) Product of RGO hydrogel (moisture ∼97%). (F) Drawing of water-intercalated structure of RGO hydrogel.

Fig. 2 presents the synthetic pathway of the oxidation and reduction reactions starting from natural graphite (Fig. 2A) to produce an RGO hydrogel structure (Fig. 2E). The oxidation synthesis using Mn(vii) to produce producing GrO has been described above (Fig. 2B). Sonication of the GrO particles in water prepared a concentrated dispersion of GO nanosheets that was used for the reduction synthesis of RGO hydrogel (Fig. 2C). While a variety of reducing agents, such as hydrazine, ascorbic acid and hydroiodic acid, are suitable to remove functional groups from GO nanosheets, it is found that ammonia solution with high alkalinity is a good reducing agent. It is well-known that an aqueous ammonia environment (at pH 10) creates an electrostatic repulsion force among negatively charged RGO nanosheets, stabilizing the RGO nanosheets in a colloidal dispersion.^39^ Additionally, the effective reduction reaction of strong alkali solutions of NaOH or KOH was reported by X. Fan and colleagues.^55^ The obtained RGO suspensions were colloidally stable in the strong alkaline environment due to electrostatic stabilization. Aqueous media with strong alkalinity are considered to be an environmentally friendly approach for synthesizing processable graphene.^56^

In our synthetic procedure, an aqueous mixture of ammonium hydroxide (ammonia concentration 2.5% with pH 14) was prepared for the chemical reduction of GO nanosheets at 90 °C (Fig. 2D). The highly alkaline ammonium hydroxide solution gave a uniform reducing effect on the GO nanosheets. The oxygen-containing functional groups on the GO nanosheets were gradually reduced during the reaction. Self-assembly of the hydrophobic RGO nanosheets occurred to form RGO agglomerates in the suspension. The remaining hydrophilic functional groups on the RGO nanosheets retained hydration layers for intercalation among the RGO nanosheets. The functional groups were negatively charged to provide electrostatic repulsion force, preventing the restacking of the RGO nanosheets. After the reduction reaction, the RGO nanosheets self-assembled into three-dimensional agglomerates, facilitating the processes of filtration and washing on filter paper. After washing and drying, a RGO hydrogel (moisture ∼97% and solid content of ∼3%) was obtained (Fig. 2E). The three-dimensional network of RGO nanosheets retained a high water content in the hydrogel structure (Fig. 2F). Like the hydrated walls of biological cells in natural plants that maintain the biological cellular structures, water molecules play an important role as intersheet spacing layers that protect the non-stacked RGO nanosheets from irreversible restacking.^41,48^

**Fig. 3:**
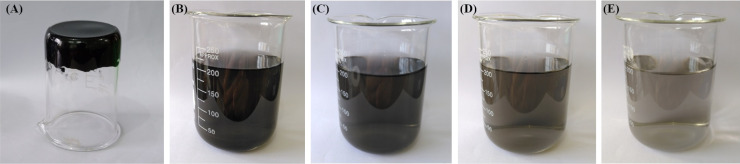
As-prepared RGO hydrogel (A) and dispersions of RGO in water at concentrations of 9 ppm (B), 7 ppm (C), 5 ppm (D) and 3 ppm (E).

Fig. 3A presents the viscoelastic property of the RGO hydrogel, which is very viscous and resists gravitational flow. The supramolecular network of intermolecular hydrogen bonding in the RGO hydrogel is responsible for the viscosity. In the ultrasonic treatment in aqueous solution, the typical water-intercalated structure of the GrO accounts for the exfoliation mechanism of multilayer GrO into GO single layers. Similarly, when the RGO hydrogel is ultrasonically dispersed in water, the transmission of ultrasound waves into the aqueous channels in the water-intercalated structure leads to water vibrations and cavitation zones, exfoliating the macroscopic hydrogel into micro-hydrogels and RGO nanosheets. Fig. 3B–E show the homogenous dispersions of RGO nanostructures in water at concentrations of 9 ppm, 7 ppm, 5 ppm and 3 ppm, respectively. The 7 ppm RGO dispersion was analyzed using transmission electron microscopy to confirm the two-dimensional nanosheets (in the analysis of transmission electron microscopy, scanning electron microscopy and elemental mapping). The aqueous dispersibility of the RGO hydrogel is a crucial advantage in processing technology. Consequently, the RGO hydrogel with three-dimensional assembly and water intercalation is a good structure that preserves non-stacked RGO nanosheets for subsequent processing and application.

### X-ray diffraction crystallography and Fourier-transform infrared spectroscopy

**Fig. 4:**
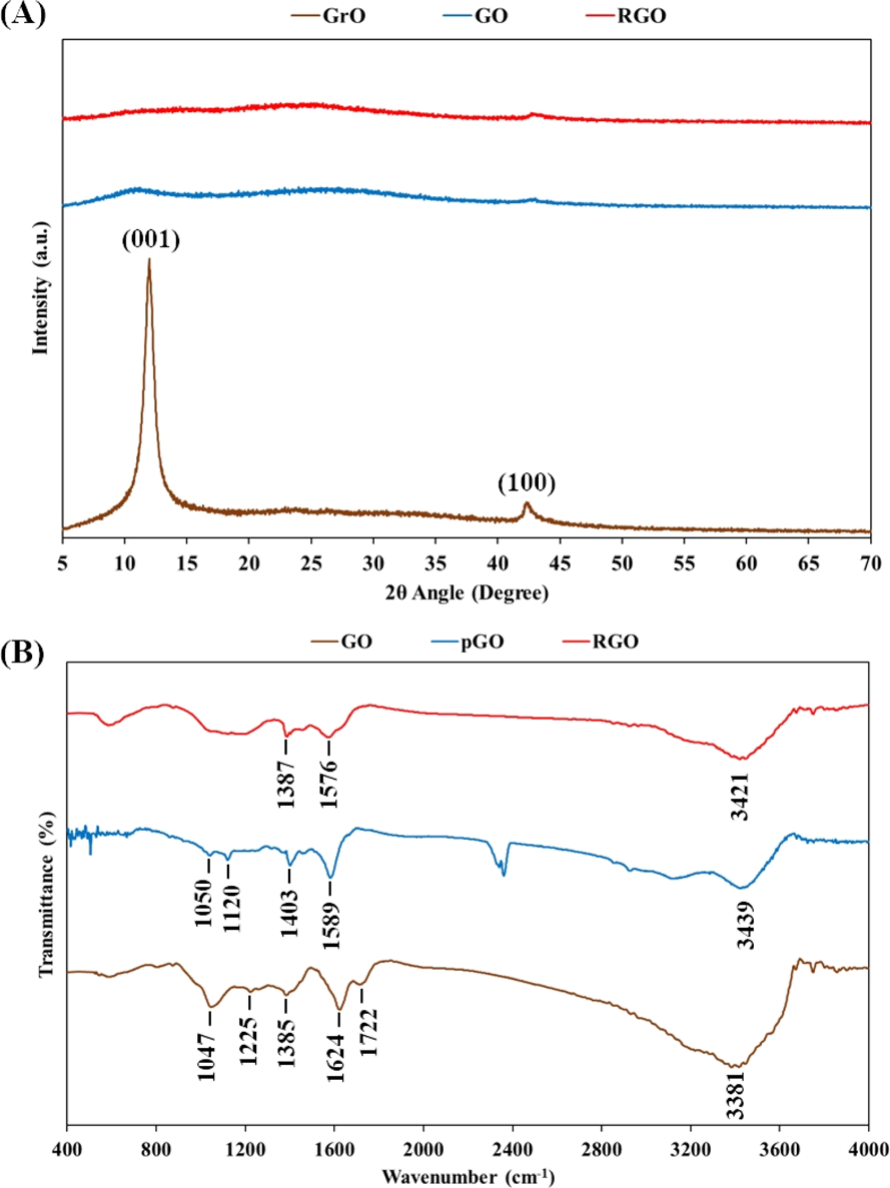
(A) XRD patterns of graphite oxide (GrO), graphene oxide (GO) and reduced graphene oxide (RGO). (B) FTIR spectra of graphene oxide (GO), partially reduced graphene oxide (pGO) and reduced graphene oxide (RGO).

The transformations of graphite into GrO, GO, pGO and RGO were characterized using XRD crystallography and FTIR spectroscopy (Fig. 4). As shown in Fig. 4A, XRD pattern of GrO powder showed a (001) peak at 11.4° and (100) peak at 43°. The shift of the (001) peak from 26.5° in graphite to 11.4° in GrO indicated that the spacing of the interlayer galleries was enlarged from 0.33 nm to 0.78 nm due to the intercalation of oxygen-containing functional groups and water layers. The intercalated water layers in the GrO structure are aqueous channels for ultrasound transmission and cavitation, leading to ultrasonic exfoliation into GO nanosheets in a water environment. The XRD pattern of the GO powder showed the disappearance of the sharp peak of the (001) lattice. The exfoliated GO nanosheets would have assembled in a random manner instead of as a layered crystal structure. After the chemical reduction reaction and oven drying, the crystal peak of the (001) lattice was not observed in the XRD pattern of the RGO powder, suggesting the random assembly of the RGO nanosheets.

The FTIR spectrum of the GO nanosheets in Fig. 4B includes peaks corresponding to hydroxyl groups (O–H stretching at 3381 cm^−1^), carboxylic groups (C–O–H bending at 1385 cm^−1^ and C=O stretching at 1722 cm^−1^), epoxide groups (C–O–C vibration at 1047 cm^−1^) and particularly the peak of covalent sulfate groups (S=O stretching at 1225 cm^−1^).^20^ Additionally, the peak at 1624 cm^−1^ is characteristic of the bending vibration of water molecules on hydrophilic GO nanosheets.^57,58^ In the next stage, the chemical reaction with highly alkaline ammonia caused the reduction of functional groups, such as carboxylic groups and epoxide groups. In fact, in the FTIR spectra of pGO and RGO, the typical carboxylic peak at 1722 cm^−1^ disappeared, and the intensity of the epoxide peak at ∼1050 cm^−1^ was also considerably reduced. However, the peaks of the hydroxyl groups (at ∼3400 cm^−1^) and aromatic C=C domains (at ∼1580 cm^−1^) are quite obvious in FTIR spectra of the pGO and RGO nanosheets. It is important that hydrophilic functional groups, typically hydroxyl groups, remain present in the RGO nanostructure, as they are responsible for hydration on the RGO nanosheets as well as water intercalation in the RGO hydrogel. The RGO nanosheets were hydrated and intercalated with water layers that prevented van der Waals stacking of the graphene-based nanosheets.

### Light absorption spectroscopy and optical band gap energy

**Fig. 5:**
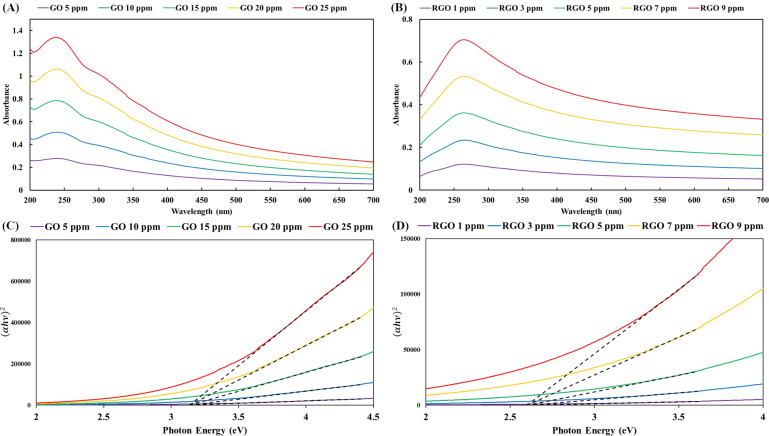
(A and B) UV-vis absorption spectra of aqueous dispersions of GO nanosheets and RGO nanosheets, respectively. (C and D) Tauc plots for the estimation of the optical band gap energy of the GO nanosheets (3.14 eV) and RGO nanosheets (2.61 eV) respectively.

Due to the water-intercalated structure of the three-dimensional hydrogels, the ultrasound treatment of RGO hydrogel in water produced homogeneous dispersions of RGO nanostructures. Aqueous dispersions with specific RGO concentrations (Fig. 3B–E) were analyzed using UV-vis spectroscopy. In the UV-vis spectra of the GO dispersions (Fig. 5A), the main absorption peak is observed at 240 nm (π–π* electronic transition of the aromatic domain) with a shoulder at 306 nm (*n*–π* electronic transition of the oxidized domain). Additionally, the UV-vis spectra of the RGO dispersions presented an obvious peak at 265 nm (Fig. 5B). Restoration of the π-conjugated network extended the aromatic domains in the RGO nanosheets, resulting in the red-shift of the light absorption peak of the π–π* electronic transition.

The UV-vis spectral data were transformed into the plots in Fig. 5C and D using the Tauc equation. The convergence of the tangential lines at a point on the horizontal axis of photon energy provides the optical band gap energy. In accordance with the Tauc plots in Fig. 5C and D, the band gap energies of the GO and RGO nanosheets were estimated to be 3.14 eV and 2.61 eV, respectively. The lower band gap energy of the RGO nanosheets is equivalent to a higher degree of reduction or better π-conjugated network in the graphene domains. Mild reduction reactions allow gradual deoxygenation and sp^2^ carbon restoration in order to tune the band gap of graphene-based nanosheets.^59^ The chemical reaction using highly alkaline ammonia at 90 °C produced different degrees of reduction with respect to the reaction time. The pGO hydrogel (reaction time of 2 hours) had a band gap of 2.88 eV (corresponding to an onset wavelength of 430.5 nm), while the RGO hydrogel (reaction time of 3 hours) gave a band gap of 2.61 eV (equivalent to an onset wavelength of 475 nm in the visible light spectrum).

### Transmission electron microscopy, scanning electron microscopy and elemental mapping

**Fig. 6:**
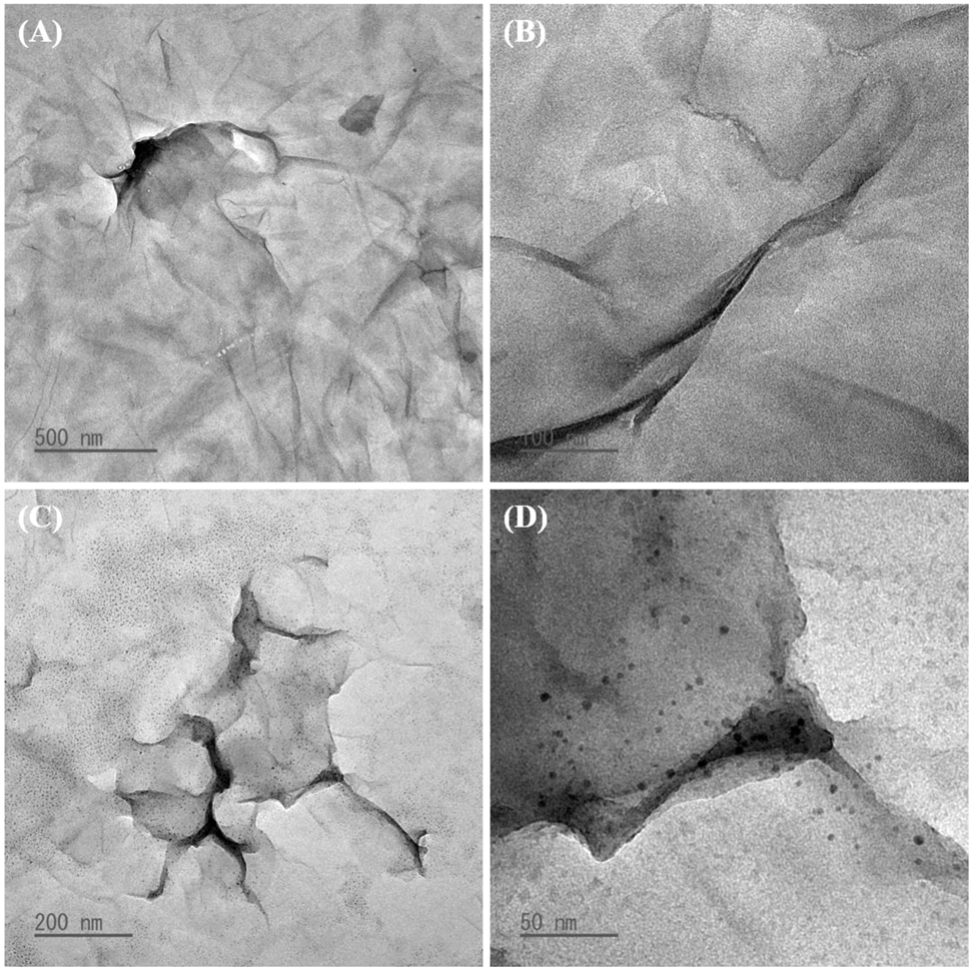
TEM images of GO nanosheets (A and B) and RGO nanosheets (C and D).

In the context of nanotechnology, the two-dimensional and elemental structures of graphene-based nanosheets are important features governing their interfacial and electronic properties for a variety of applications. The TEM technique is one of the most authentic methods to confirm the nanostructures of graphene-based nanosheets. Fig. 6A and B reveal the nano-layers, wrinkles and edges of the GO nanosheets. The RGO hydrogel synthesized in this study was sonicated in water to produce a homogeneous dispersion of RGO nanosheets (concentration ∼7 ppm, Fig. 3C) for TEM analysis. As shown in Fig. 6C and D, the TEM images of RGO nanosheets revealed the shape and size of single-layer and few-layer RGO nanosheets. Similar to water-intercalated structure of GrO, the three-dimensional structure of the RGO hydrogel is intercalated with water layers that prevent graphene stacking and vibrate with ultrasound waves to create cavitations for RGO exfoliation in the water medium. The TEM visualization demonstrated that the RGO hydrogel was ultrasonically exfoliated into RGO nanosheets.

**Fig. 7:**
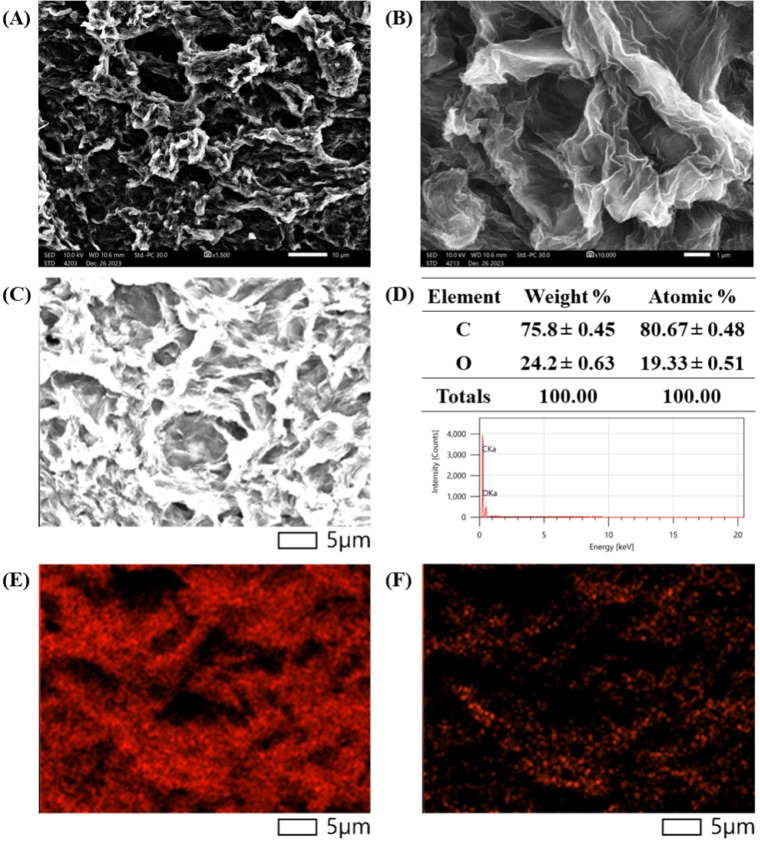
(A and B) SEM images of the three-dimensional assembly of the RGO hydrogel with scale bars of 10 μm and 1 μm, respectively. (C) SEM image of the RGO structure in EDS analysis. (D) EDS spectrum and elemental composition of carbon and oxygen in the RGO structure. (E and F) Elemental mapping of carbon atoms and oxygen atoms, respectively, on the RGO structure.

The morphological structure and elemental composition of the RGO hydrogel were analyzed using the integrative method of SEM and EDS analyses. In Fig. 7A and B, the SEM images present the porous morphology of three-dimensional assembly of the RGO nanosheets derived from the RGO hydrogel. Although RGO hydrogel was dried to evaporate water and some RGO nanosheets stacked to form a self-assembled structure, the porous morphology indicated three-dimensional assembly and water intercalation in the RGO hydrogel. As shown in Fig. 7C and D, EDS analysis revealed that the RGO nanostructure was composed of 80.67% carbon atoms and 19.33% oxygen atoms. The C/O atomic ratio of 4.17 is comparable with that of RGO reduced by hydrazine (C/O ratios in the range of 2.67–4.44, Table 2) and lower than the value of RGO obtained from the reduction reaction using hydroiodic acid (C/O ratio of 5.6, Table 2).^60–62^Fig. 7E and F exhibit the elemental mapping of carbon atoms and oxygen atoms, respectively, on the RGO nanostructure. The general distribution of oxygen atoms on the RGO nanosheets implies the presence of oxygen-containing functional groups that retain hydration layers as spacing intercalants in the RGO hydrogel.

**Table 2:** Atomic percentages, C/O ratio and optical band gap energy of graphene-based materials

Material	C (%)	O (%)	C/O ratio	Band gap (eV)
GO nanosheets^a^	61.05	37.76	1.62	3.14
pGO hydrogel^a^	72.45	27.55	2.63	2.88
RGO hydrogel^a^	80.67	19.33	4.17	2.61
RGO from hydrazine reduction^60^	81.61	18.39	4.44	
RGO from hydroiodic acid reduction^61^	80.68	14.41	5.6	
RGO from hydrazine reduction^62^	72.76	27.24	2.67	
RGO from multiphase reduction^62^	78.3	21.7	3.61	
RGO obtained by hydrazine reduction^63^	73.13	26.87	2.68	2.72
RGO obtained by voltage application^63^	80.02	19.92	4.02	2.54

a Materials synthesized in this work. Elemental compositions were analyzed using EDS method.

Table 2 summarizes the C/O atomic ratios and band gap values of the various graphene-based nanomaterials. As the degree of reduction of the graphene nanosheets increases, the C/O ratio becomes higher and the band gap energy becomes lower. The reduction degree of the RGO hydrogel is considered to be sufficient with a C/O ratio of 4.17 and a band gap energy of 2.61 eV. In comparison with the scientific literature, the reduction degree of the RGO hydrogel is better than that of hydrazine-reduced RGO (C/O ratio of 2.68 and band gap of 2.72 eV) and voltage-reduced RGO (C/O ratio of 4.02 and band gap of 2.54 eV).^63^ In the investigation by N. Morimoto *et al.*, it was found that an RGO nanomaterial with an oxygen weight content of 23.1% (oxygen atomic content of 18.39% and C/O ratio of 4.44) gave the best supercapacitor electrode performance.^60^ RGO nanosheets with sufficient oxygen-containing functional groups achieved an optimal balance between large surface area and high electrical conductivity. Therefore, the chemical reduction method using highly alkaline ammonia produced a good degree of reduction of the RGO nanosheets (oxygen atomic percentage of 19.33% and C/O ratio of 4.17) for electrochemical applications.

### Particle size analysis and water purification application

**Fig. 8:**
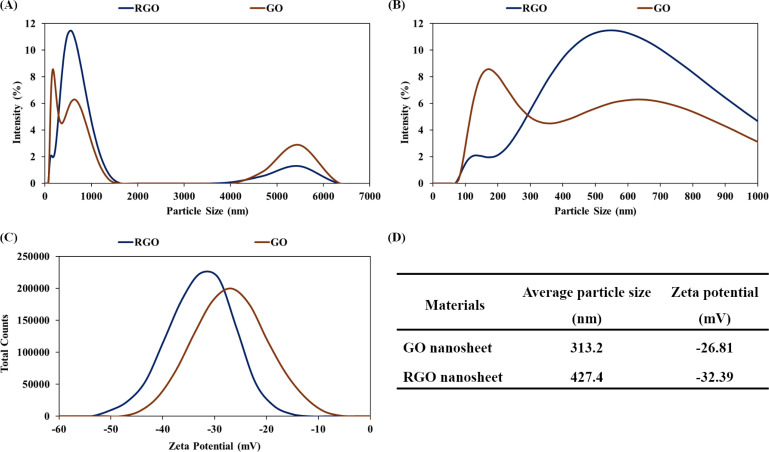
(A) Particle size analysis of GO and RGO nanosheets in water solutions (concentration of 7 ppm). (B) Expended view of the particle size distribution in the range of 0–1000 nm. (C) Graph of the zeta potentials of the GO and RGO nanosheets. (D) Summary of average particle size and zeta potential of the GO and RGO nanosheets.

Reversible self-assembly is a solution-processable property of graphene-based hydrogel.^48,49^ The GO nanosheets were chemically reduced and self-assembled into a hydrogel structure of RGO nanosheets. Water intercalation in the RGO hydrogel facilitated the ultrasonic exfoliation of the three-dimensional assembly into micro-hydrogels and nanostructures. Dilute dispersions of GO and RGO nanosheets in water (concentration of 7 ppm) were analyzed using the dynamic light scattering and zeta potential methods. Fig. 8A and B show the particle size distributions of the GO and RGO nanosheets in water solutions at a neutral pH of 7. Because GO and RGO are two-dimensional nanosheets, the proportion of particle sizes in the range of 4–6 μm would be assigned to the width of the nanosheets. Major proportions of particle size in the range below 1 μm would include the thickness dimension and leaning orientation of GO and RGO nanosheets. Fig. 8C presents the curves of the zeta potentials of the GO and RGO nanosheets in the aqueous solutions. As summarized in Fig. 8D, the average particle sizes of GO and RGO were 313.2 nm and 427.4 nm, respectively. These average values are good means for evaluating the dispersions of RGO nanosheets in water.^39^ Herein, the average particle size of RGO nanosheet (427.4 nm) was comparable with that of the GO nanosheets (313.2 nm), indicating that the RGO nanostructures were well-exfoliated and dispersed in water. The RGO hydrogel structure protected the RGO nanosheets from irreversible stacking, leading to the ultrasonic dispersion of RGO nanostructures in water. In addition, the zeta potential of the RGO nanosheets was determined to be −32.39 mV. This zeta potential was below −30 mV, indicating the colloidal stability of the RGO dispersion. The chemical reduction by alkaline ammonia solution made the RGO nanosheets negatively charged, which was responsible for the electrostatic repulsion and colloidal dispersion of the RGO nanosheets. Well-dispersed RGO nanosheets in water are versatile building blocks for nanotechnological applications, such as preparations of RGO coatings and polymer/RGO nanocomposites.^64^

**Fig. 9:**
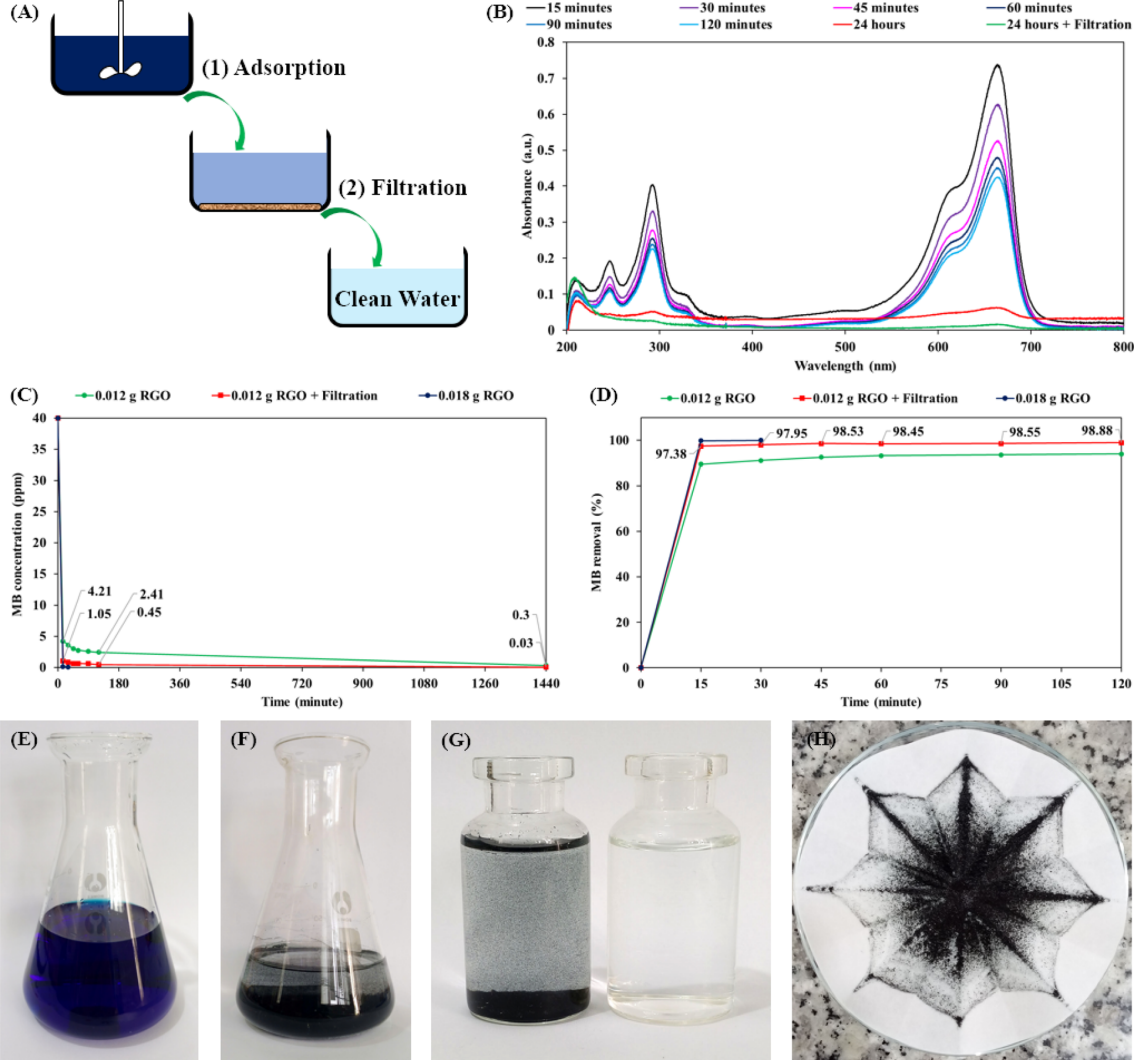
Water purification experiments. (A) Scheme of the water purification processes in the experiment, including (1) dye adsorption by RGO hydrogel and (2) filtration through ordinary cellulose paper into a reservoir of clean water. (B) UV-vis spectra of MB solutions after specific periods of the purification process using 0.012 g RGO (0.4 g RGO hydrogel). (C) Graph of MB concentration with respect to adsorption time. (D) Graph of MB removal performance with respect to adsorption time. (E) Initial MB solution at a concentration of 40 ppm. (F) MB/RGO suspension after agitation of 200 mL of 40 ppm MB solution and 0.6 g RGO hydrogel (0.018 g RGO) for 1 hour. (G) MB/RGO suspension before filtration (left bottle) and the obtained clean water after filtration (right bottle). (H) MB/RGO solid remaining on cellulose paper after the filtration process.

In the next experiment, the RGO hydrogel product was applied for water purification (the adsorption and removal of MB dye molecules in water). As shown in Fig. 9A, RGO hydrogel was dispersed in 40 ppm MB solution using agitation for a specified time period to achieve MB adsorption, followed by filtration through ordinary cellulose paper. Fig. 9B presents the UV-vis spectra of the MB solutions; the decline in the intensity of the absorption peaks at 665 nm is due to the adsorption of MB on 0.012 g RGO after water treatment process for the indicated times. The graph in Fig. 9C shows the decrease in the MB concentration as a function of adsorption time for different RGO amounts and treatment processes. Accordingly, with a 0.4 g dose of RGO hydrogel (0.012 g RGO), the MB concentration dropped from 40 ppm to 4.21 ppm after 15 minutes of adsorption. The MB concentration then gradually decreased to 2.41 ppm at an adsorption time of 120 minutes and 0.3 ppm at an adsorption time of 1440 minutes (24 hours). It is notable that filtration through cellulose filter paper further decreased the MB concentration. As a result, the treatment process combining 24 hours of adsorption and subsequent filtration reduced the MB concentration as low as 0.03 ppm. When 0.6 g RGO hydrogel (0.018 g RGO) was dispersed in 200 mL of 40 ppm of MB solution, the adsorption process was much faster. The MB concentration dropped to 0.11 ppm after 15 minutes and reached 0.03 ppm in only 30 minutes. Fig. 9D presents the MB removal performances over a period of 120 minutes in detail. The adsorption of MB on the RGO structures was fastest in the first 15 minutes and last 24 hours. Using a 0.4 g dose of RGO hydrogel (0.012 g RGO), the process combining 24 hours of adsorption and subsequent filtration produced clean water (MB removal performance of 99.93%). With the 0.6 g dose of RGO hydrogel (0.018 g RGO), the 30 minute adsorption process was sufficient to attain an MB removal of 99.93%.

Fig. 9E and F depict the initial 40 ppm MB solution and the MB/RGO suspension after 1 hour of agitation, respectively. Fig. 9G shows the MB/RGO suspension (left bottle) that was filtered through cellulose paper to obtain clean water (right bottle). Since the agitation did not fully exfoliate the RGO hydrogel in the MB solution, ordinary cellulose filter paper (pore size of 15–20 μm) easily retained the MB/RGO macrostructures (Fig. 9H). A small amount of RGO hydrogel (0.6 g hydrogel, equivalent to 0.018 g RGO) purified 200 mL of 40 ppm MB solution, producing clean water *via* a facile approach. The MB adsorption capacity of the RGO hydrogel was estimated to be 666.2 mg g^−1^, which classifies it as an excellent material for organic dye adsorption.^48,65^ For comparison, recent research has indicated that the MB adsorption capacities of activated carbon materials are in the range of 269.3–996.5 mg g^−1^ owing to their microporous structures.^66–71^ The MB adsorption of the RGO hydrogel is higher than the values for graphene-based materials in a number of scientific papers,^72–74^ and is comparable with the best value of RGO materials reported by B. Nissanka *et al.* (∼700 mg g^−1^).^75^ The high MB adsorption performance of the as-synthesized RGO hydrogel is attributed to the supramolecular structure and large surface area of the RGO nanosheets. In the water purification process involving adsorption and filtration, our filtration step used inexpensive cellulose filter paper (pore size of 15–20 μm) to attain a very fast flow rate of 7.032 mL min^−1^ (∼84 L m^−2^ h^−1^). The processes of agitation and filtration are simple, cost-effective and applicable for practical water purification in households and industry. Organic dye molecules adsorbed on the RGO structures can be desorbed in ethanol, regenerating the RGO hydrogel for next adsorption application.

## Conclusion

The synthetic scheme of oxidation–reduction reactions is a principal pathway for synthesizing graphene-based nanosheets from natural graphite. It is essential to improve scientific mechanisms and technological innovations for prospective production and applications. The cascade-design oxidation reaction using Mn(vii) is an appropriate approach to harness exothermic energy and optimize chemical and energy efficiencies. The reaction temperature trajectories were investigated to control the thermal runaway process and elucidate the self-heating reaction mechanisms for safe and efficient production. The reaction of dilute hydrogen peroxide solution had a positive effect on the degree of oxidation and solid yield of GrO product. In the next stage, chemical reduction reaction, it was found that highly alkaline ammonium hydroxide solution is an effective reducing environment for synthesizing a three-dimensional hydrogel of water-intercalated RGO nanosheets. The obtained RGO nanosheets have a good degree of reduction with an optimized C/O ratio of 4.17 and optical band gap of 2.61 eV. Considerable amounts of oxygen-containing functional groups were present to retain hydration layers on the RGO nanosheets. Inspired by biological hydrated cellular walls and artificial hydrated multilayer graphitic oxide, the three-dimensional assembly and water intercalation in the RGO hydrogel structure are important to preserve non-stacked RGO nanosheets. As ultrasound waves were transmitted into the aqueous channels in the RGO hydrogel, the macroscopic hydrogel was ultrasonically exfoliated into nanostructures in water. In water purification applications, the facile dispersion and high performance of RGO hydrogel in the treatment of organic dye solution open opportunities for practical environmental remediation. The three-dimensional hydrogel of water-intercalated RGO nanosheets is a promising concept for graphene nanotechnology.

## Data availability

The data supporting this article have been included as part of the ESI† and deposited at Data 20/07/2024 at https://drive.google.com/drive/folders/1vvAJelZi7PFPrrGNy10yTm8wJKM0A6am?usp=sharing.

## Author contributions

H. N. Le: conceptualization, methodology, experimentation, investigation, writing – review and editing. T. B. T. Dao, T. D. Nguyen and D. A. Dinh: analytical support and manuscript review. C. N. Ha Thuc and V. H. Le: project supervision and manuscript review.

## Conflicts of interest

There are no conflicts of interest to declare.

## Supplementary Material

RA-014-D4RA05385K-s001
