# Supplementary material for: Revisiting oxidation and reduction reactions for synthesizing a three-dimensional hydrogel of reduced graphene oxide
Source: RSC Adv. 2024 Sep 27;14(42):30844–58. doi: 10.1039/d4ra05385k (PMC11427890; doi:10.1039/d4ra05385k)
Supplement: RA-014-D4RA05385K-s001 [file RA-014-D4RA05385K-s001.pdf]

Supplementary Information (SI) for RSC Advances.
This journal is © The Royal Society of Chemistry 2024

# Electronic Supplementary Information

# Revisiting Oxidation and Reduction Reactions for Synthesizing Three Dimensional Hydrogel of Reduced Graphene Oxide

**Hon Nhien Le,*[ac] Thi Bang Tam Dao,[ac] Trung Do Nguyen,[ac] Duc Anh Dinh,[d] Chi Nhan Ha Thuc *[ac] and Van Hieu Le *[abc]**

[a] Faculty of Materials Science and Technology, University of Science, 227 Nguyen Van Cu Street, Ward 4, District 5, Ho Chi Minh City, 700000, Vietnam. Emails: lhnhien@hcmus.edu.vn, htcnhan@hcmus.edu.vn, lvhieu@hcmus.edu.vn

[b] Multifunctional Materials Laboratory, University of Science, Ho Chi Minh City, 700000, Vietnam

[c] Vietnam National University, Linh Trung Ward, Thu Duc City, Ho Chi Minh City, 700000, Vietnam

[d] NTT Hi-Tech Institute, Nguyen Tat Thanh University, Ho Chi Minh City, 700000, Vietnam

## 1. Investigation of temperature trajectory of graphite oxidation reaction

In the investigation of graphite oxidation reaction, two reaction processes were conducted and analyzed with real-time temperature measurements by an infrared thermometer. Table S1 presents the time – temperature data of the reaction (1) and the reaction (2). The reaction time began (at t = 0 minute) when $KMnO_4$ was dissolved in $H_2SO_4$ 96% to prepare the oxidizing solution of Mn(VII)/$H_2SO_4$. In the first cascade step, the suspension of graphite/$H_2SO_4$ was poured slowly into the Mn(VII)/$H_2SO_4$ solution (at t = 60 minutes). The reactor (1) was put in an ambient water bath (water temperature ~ 28 $^oC$) for 5 minutes (from t = 60 minutes to t = 65 minutes), while the reactor (2) was cooled in an ambient water bath for 10 minutes (from t = 60 minutes to t = 70 minutes). Then, the reactors were magnetically stirred in ambient room condition. Significant increases of reaction temperatures resulted from the intercalation and reaction of Mn(VII)/$H_2SO_4$ compound in graphite structures.

In the second cascade step (at t = 300 minutes), the mixture of graphite/Mn(VII)/$H_2SO_4$ was gradually poured into a glass beaker of water under magnetic stirring. Intense exothermic processes were obsersed to elevate reaction temperatures to more than 90 $^oC$ with the heating speed of ~ 21 $^oC$/minute. The hydration reaction of concentrated sulfuric acid and water generated strongly exothermic heat. Suitable amount of water in the process absorbed the exothermic heat to keep the reaction safe and efficient. After the exothermic peak, the reaction temperature naturally decreased to ambient room condition. Exothermic energy of the self-heating process is sufficient for graphite oxidation reaction, which is important to save heating energy in industrial production.

The addition of hydrogen peroxide solution 5 % to the reaction mixture was carried out at t = 420 minutes. Noticeable reaction temperature increase as well as exothermic process was recorded.

Table S1. Statistic of reaction temperature with respect to synthetic process time (corresponding to Figures 1A and 1B in the main manuscript).

| Time (minute) | Temperature of reaction (1) ($^oC$) | Temperature of reaction (2) ($^oC$) | Note |
|---|---|---|---|
| 0 | 28 | 29 | Exothermic heat of dissolution of |
| 5 | 30 | 32 | |

| | | | |
|---|---|---|---|
| 10 | 31 | 34.5 | $KMnO_4$ in $H_2SO_4$ |
| 15 | 34 | 33.8 | |
| 20 | 32.8 | 32.8 | |
| 25 | 31.9 | 32.3 | |
| 30 | 30.9 | 31.8 | |
| 35 | 30.4 | 31.7 | |
| 40 | 30.1 | 31.6 | |
| 45 | 29.8 | 31.1 | |
| 50 | 29.3 | 30.7 | |
| 55 | 29 | 30.6 | |
| 60 | 29 | 30.5 | Exothermic reaction in the first cascade step |
| 65 | 32.6 | 30.5 | |
| 70 | 41.5 | 34.5 | |
| 75 | 52 | 47.6 | |
| 80 | 48.4 | 51.2 | |
| 85 | 44.9 | 46.7 | |
| 90 | 42.7 | 44.2 | |
| 95 | 40.4 | 42 | |
| 100 | 38.7 | 40.5 | |
| 105 | 37.5 | 39.3 | |
| 110 | 36.3 | 38.6 | |
| 115 | 35.1 | 37.3 | |
| 120 | 34.2 | 36.9 | |
| 150 | 31.7 | 34 | |
| 180 | 30.3 | 32.5 | |
| 210 | 30.2 | 32.3 | |
| 240 | 30.1 | 31.7 | |
| 270 | 30 | 31.6 | |
| 300 | 30 | 31.5 | Exothermic reaction in the second |
| 301 | 71 | 49.5 | |

| | | | |
|---|---|---|---|
| 302 | 95.4 | 75 | cascade step |
| 303 | 94 | 93 | |
| 304 | 90.5 | 91 | |
| 305 | 87.5 | 87.3 | |
| 306 | 85.3 | 84 | |
| 307 | 82.7 | 83.8 | |
| 308 | 80.9 | 79.7 | |
| 309 | 79.1 | 77 | |
| 310 | 76.3 | 75.2 | |
| 311 | 74.2 | 73.9 | |
| 312 | 73.6 | 71.4 | |
| 313 | 72.3 | 71 | |
| 314 | 70.7 | 70 | |
| 315 | 69.7 | 69.1 | |
| 320 | 63.5 | 63.2 | |
| 325 | 59.1 | 59.1 | |
| 330 | 55.7 | 55.4 | |
| 335 | 52.6 | 52.3 | |
| 340 | 49.8 | 50.4 | |
| 345 | 47.8 | 48.5 | |
| 350 | 45.2 | 46.5 | |
| 355 | 43.5 | 44.6 | |
| 360 | 42.2 | 43.7 | |
| 390 | 36 | 37.9 | |
| 420 | 33.1 | 34.5 | Exothermic heat of addition of $H_2O_2$ 5% to the reaction |
| 421 | 35 | 36.2 | |
| 422 | 34.8 | 36.2 | |
| 423 | 34.6 | 36.1 | |
| 424 | 34.4 | 35.8 | |
| 425 | 34.2 | 35.7 | |

| | | | |
|---|---|---|---|
| 430 | 33.8 | 35 | |
| 435 | 33.5 | 34.5 | |
| 440 | 33.1 | 34 | |
| 445 | 33 | 33.5 | |
| 450 | 32.9 | 33 | |

**2. Calculation of exothermic heat of the first cascade step in the reaction (1) and reaction (2)**

The first cascade step was investigated in the reaction (1) (cooling time in an ambient water bath is 5 minutes) and in the reaction (2) (cooling time in an ambient water bath is 10 minutes). After the cascade step, the reaction temperature gradually increased because of thermal runaway reaction. Heat released from the exothermic reaction can be calculated using the equation:

$$\Delta H = \Delta T \times C_p \times M_R$$

where ΔH is the exothermic heat of reaction (KJ), ΔT is the temperature increase, $M_R$ is the mass of the reaction mixture and the reactor (kg), $C_p$ is the heat capacity of the reactor mixture (KJ $kg^{-1}$ $K^{-1}$). Mass of the reactor mixute in the experiments is 0.4307 kg. In accordance with scientific literature, the value of 1.92 KJ $kg^{-1}$ $K^{-1}$ is referenced as the heat capacity for estimated calculation.[1]

Table S2. Summary of exothermic heat and power of the first cascade step in reaction (1).

| Time (minute) | Temperature increase (°C) | Exothermic heat (KJ) | Exothermic power (W) |
|---|---|---|---|
| 60 | 0 | 0 | 0 |
| 65 | 3.6 | 2.98 | 9.92 |
| 70 | 8.9 | 7.36 | 24.53 |
| 75 | 10.7 | 8.85 | 29.49 |

Table S3. Summary of exothermic heat and power of the first cascade step in reaction (2).

| Time (minute) | Temperature increase ($^oC$) | Exothermic heat (KJ) | Exothermic power (W) |
|---|---|---|---|
| 65 | 0 | 0 | 0 |
| 70 | 4 | 3.31 | 11.03 |
| 75 | 13.1 | 10.83 | 36.11 |
| 80 | 3.6 | 2.98 | 9.92 |

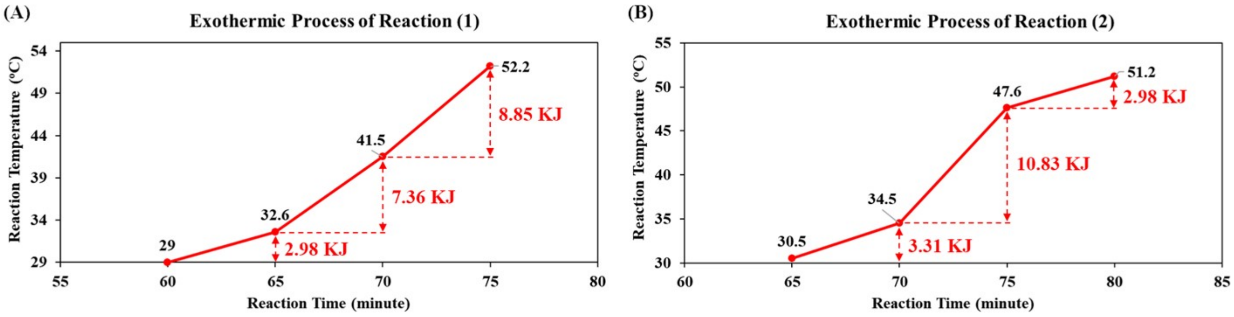


Figure S1. Calorimetry diagrams of the exothermic processes of the first cascade step in the reaction (1) and the reaction (2).

According to Figure S1, total heats released from the exothermic process in the reaction (1) and the reaction (2) are 19.19 KJ and 17.12 KJ respectively. The total exothermic heat in the reaction (2) is reduced by 10.79 % compared to the value of the reaction (1). The lower exothermic heat in the reaction (2) is explained due to the longer cooling time in the ambient water bath that dissipated a part of exothermic heat, leading to the smaller increase of reaction temperature.

**3. Dispersions of graphene oxide nanosheets in water for ultraviolet-visible absorption spectroscopy and optical band gap calculation**

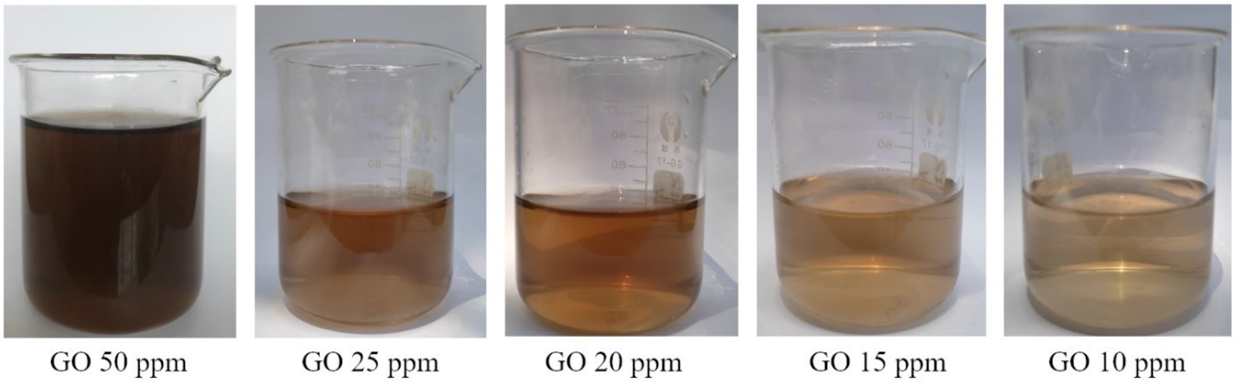


Figure S2. Aqueous disperions of graphene oxide nanosheets at the concentrations of 50 ppm, 25 ppm, 20 ppm, 15 ppm and 10 ppm.

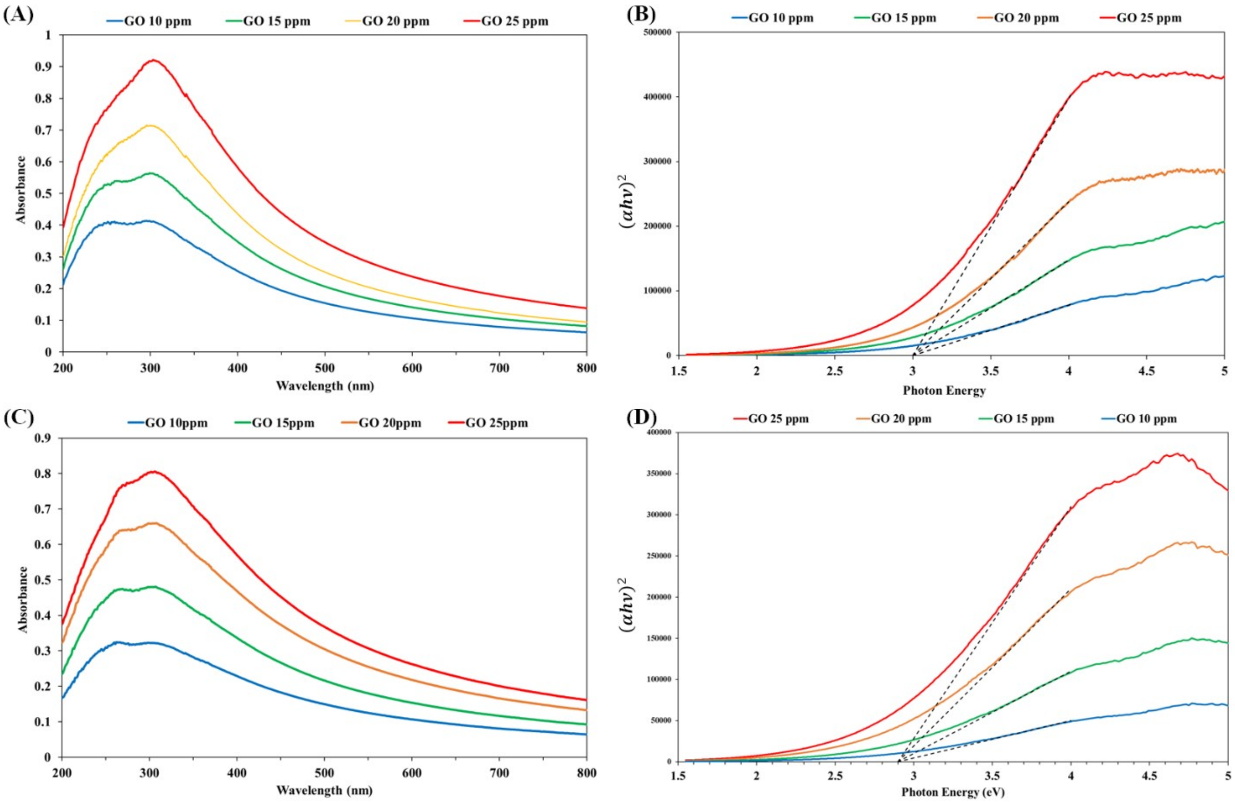


Figure S3. (A) UV-Vis spectra of GO solutions (corresponding to procedure 2 in Table 1 in the main manuscript) and the Tauc plot for band gap estimation ~ 3 eV (B). (C) UV-Vis spectra of GO solutions (corresponding to procedure 3 in Table 1 in the main manuscript) and the Tauc plot for band gap estimation ~ 2.9 eV (D).

Tauc equation and plotting method for calculating optical band gap are adapted from previous research papers.[2,3] UV-Vis spectra of GO solutions at different concentrations (10 ppm, 15 ppm, 20 ppm and 25 ppm) were transformed into Tauc plots. Tangents of the Tauc-plot curves intersect the horizontal axis at the optical band gap energy. The convergence of many tangents derived from many concentrations of GO solutions enhances the accuracy of the calculation method. Therefore, optical band gaps of different types of GO nanosheets can be determined. GO nanosheets with higher degree of oxidation has the higher band gap of 3 eV (GO produced by synthetic procedure 2), and GO nanosheets with lower amount of oxygen-containing functional groups gives the smaller band gap of 2.9 eV (GO produced by synthetic procedure 3). Actually, GO nanosheets with the highest oxidation degree as well as optical band gap (3.14 eV) were synthesized by procedure 1 (150 mL $H_2O_2$ solution 5 % was used in the oxidation reaction).

**4. Microscopic analysis of three-dimensional structure of reduced graphene oxide hydrogel**

Restacking of graphene nanosheets has been the challenging issue that causes significant declines of accessible surface area and colloidal dispersibility of graphene-based materials. $\pi$-$\pi$ interaction and van der Waals attraction forces are responsible for irrevesible stacking of graphene-based nanosheets. Van der Waals attraction potential can be described by the equation:

$$E \approx \frac{S}{d^4}$$

where E is the van der Waals attraction potential, d is the distance between graphene nanosheets, and S is the interacting surface area of graphene nanosheets.[4]

With exceptionally large surface area, van der Waals attraction potential of graphene nanosheets in close interlayer distance becomes very high, resulting in irreversible stacking. Many approaches to preventing the restacking have been reported in scientific literature, including decreasing the interacting surface area (S) and increasing the interlayer distance (d). Especially, reversible self-assembly of RGO/$SnO_2$ hydrogel was demonstrated to prevent the restacking of graphene-based nanosheets and utilize the extraordinary potential of graphene-based nanosheets.[5] Water intercalation in the three-dimensional structure effectively increases the interlayer spacing and decreases the interacting surface area.

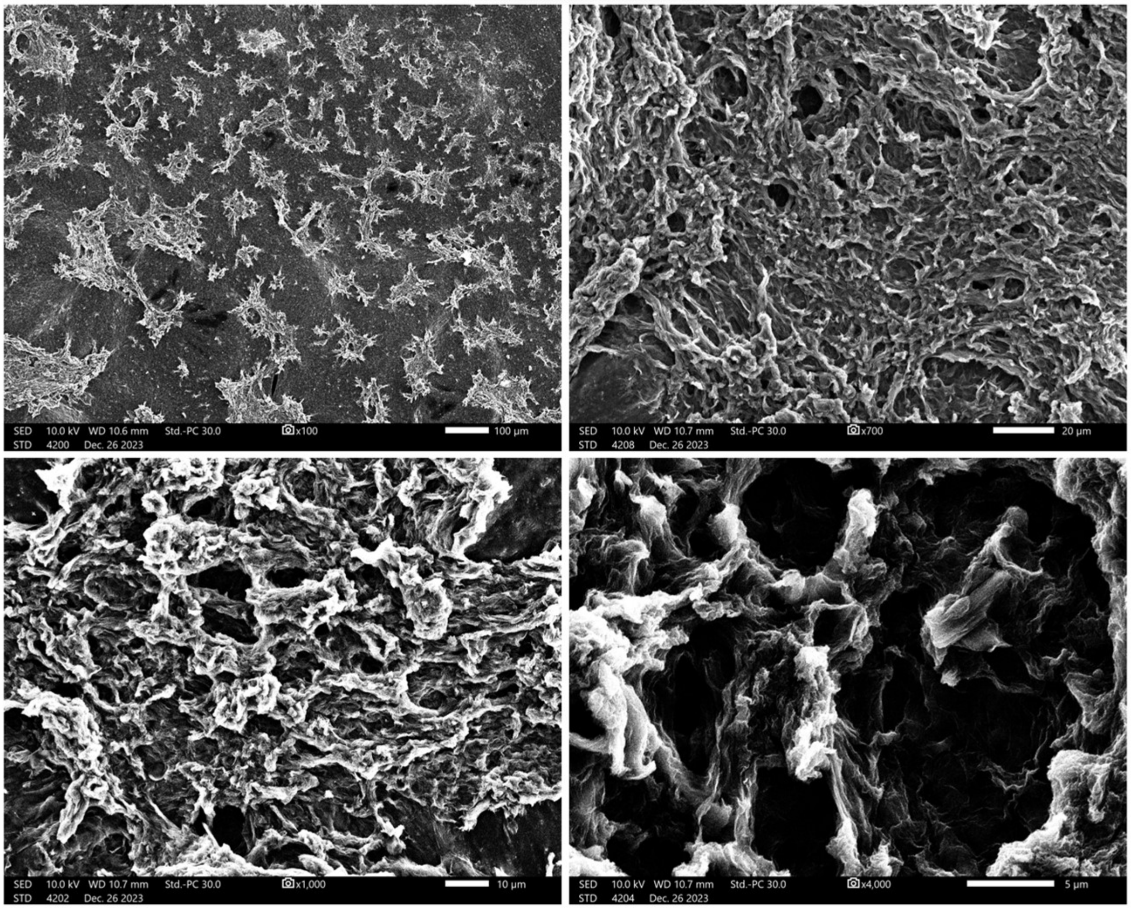


Figure S4. SEM images of RGO hydrogel spread on a carbon tape with the scale bars of 100 µm (A), 20 µm (B), 10 µm (C) and 5 µm (D).

Figure S4 presents the porous morphology of RGO micro-hydrogels prepared in this research. Three-dimensional assembly of RGO nanosheets reduces the interacting surface area and enhances the accessible surface area. In addition, water intercalation in RGO hydrogel is important to increase the interlayer spacing. The hydration of RGO nanosheets in the hydrogel structure is biomimetic to the hydration of cellular walls in biological organisms. Natural cellular structures contain high amount of water for maintaining the biological shapes and functions.[6] Therefore, RGO hydrogel is a bio-inspired structure for preventing irreversible stacking and utilizing extraordinary properties of graphene-based nanosheets.

**5. Transformation of graphene oxide nanosheets into reduced graphene oxide nanosheets**

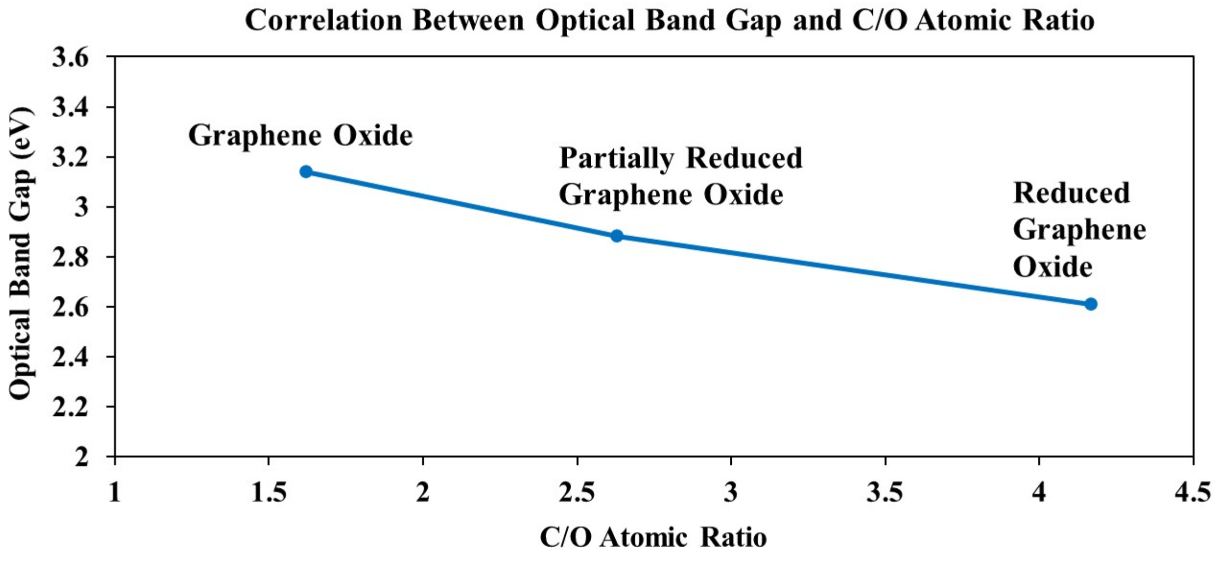


Figure S5. Presentation of optical band gap and C/O atomic ratio of GO, pGO, RGO nanomaterials synthesized by chemical reduction reaction of highly alkaline amonia solution (corresponding to Table 2 in the main manuscript).

From optical band gap energy, π-conjugated $sp^2$-carbon networks in graphene-based nanosheets can be estimated using the equation:

$$E_g = \frac{6}{\sqrt{M}}$$

where $E_g$ is the band gap energy and M is the number of six-fold aromatic rings.[7]

Accordingly, the numbers of $sp^2$-hybridized benzene rings of as-synthesized GO, pGO and RGO nanosheets are 3.65, 4.34 and 5.29 respectively. During the chemical reduction reaction using highly alkaline ammonia (pH 14), oxygen-containing functional groups on GO nanosheets were gradually reduced with respect to reaction time. As a result, the areas of π-conjugated domains in graphene-based nanosheets became larger and larger (the number of aromatic rings increased from 3.65 to 5.29). The restoration of π-conjugated networks leads to smaller band gap energy and better electrical conductivity in RGO nanosheets.

**References**

1 P. Lakhe, D. L. Kulhanek, X. Zhao, M. I. Papadaki, M. Majumder and M. J. Green, Graphene oxide synthesis: reaction calorimetry and safety, *Ind. Eng. Chem. Res.*, 2020, **59**, 9004–9014.

2 N. Sharma, M. Arif, S. Monga, M. Shkir, Y. K. Mishra and A. Singh, Investigation of bandgap alteration in graphene oxide with different reduction routes, *Appl. Surf. Sci.*, 2020, **513**, 145396.

3 H. N. Le, D. Thai, T. T. Nguyen, T. B. T. Dao, T. D. Nguyen, D. T. Tieu and C. N. Ha Thuc, Improving Safety and Efficiency in Graphene Oxide Production Technology, *J. Mater. Res. Technol.*, 2023, **24**, 4440-4453.

4 J. Luo, J. Kim and J. Huang, Material Processing of Chemically Modified Graphene: Some Challenges and Solutions, *Acc. Chem. Res.*, 2013, **46** (10), 2225–2234.

5 N. H. Le, H. Seema, K. C. Kemp, N. Ahmed, J. N. Tiwari, S. Park and K. S. Kim, Solution-processable conductive micro-hydrogels of nanoparticle/graphene platelets produced by reversible self-assembly and aqueous exfoliation, *J. Mater. Chem. A*, 2013, **1**, 12900.

6 M. Sargen and D. Utter (2019) Biological Roles of Water: Why is water necessary for life? https://sitn.hms.harvard.edu/uncategorized/2019/biological-roles-of-water-why-is-water-necessary-for-life/. Accessed 03 January 2024.

7 K. Bhardwaj, R. Kumar, N. J. Kindo, N. Vashistha, A. K. Patel, M. Kumar and P. Kumar, Synthesis of graphene oxide with a lower band gap and study of charge transfer interactions with perylenediimide, *New J. Chem.*, 2020, **44**, 12704-12714.
